# Experimental evidence of formation of transparent exopolymer particles (TEP) and POC export provoked by dust addition under current and high *p*CO_2_ conditions

**DOI:** 10.1371/journal.pone.0171980

**Published:** 2017-02-17

**Authors:** Justine Louis, Maria Luiza Pedrotti, Frédéric Gazeau, Cécile Guieu

**Affiliations:** Laboratoire d'Océanographie de Villefranche, Sorbonne Universités, UPMC University Paris 06, INSU-CNRS, Villefranche-sur-mer, France; University of Connecticut, UNITED STATES

## Abstract

The evolution of organic carbon export to the deep ocean, under anthropogenic forcing such as ocean warming and acidification, needs to be investigated in order to evaluate potential positive or negative feedbacks on atmospheric CO_2_ concentrations, and therefore on climate. As such, modifications of aggregation processes driven by transparent exopolymer particles (TEP) formation have the potential to affect carbon export. The objectives of this study were to experimentally assess the dynamics of organic matter, after the simulation of a Saharan dust deposition event, through the measurement over one week of TEP abundance and size, and to evaluate the effects of ocean acidification on TEP formation and carbon export following a dust deposition event. Three experiments were performed in the laboratory using 300 L tanks filled with filtered seawater collected in the Mediterranean Sea, during two ‘no bloom’ periods (spring at the start of the stratification period and autumn at the end of this stratification period) and during the winter bloom period. For each experiment, one of the two tanks was acidified to reach pH conditions slightly below values projected for 2100 (~ 7.6–7.8). In both tanks, a dust deposition event of 10 g m^-2^ was simulated at the surface. Our results suggest that Saharan dust deposition triggered the abiotic formation of TEP, leading to the formation of organic-mineral aggregates. The amount of particulate organic carbon (POC) exported was proportional to the flux of lithogenic particles to the sediment traps. Depending on the season, the POC flux following artificial dust deposition ranged between 38 and 90 mg m^-2^ over six experimental days. Such variability is likely linked to the seasonal differences in the quality and quantity of TEP-precursors initially present in seawater. Finally, these export fluxes were not significantly different at the completion of the three experiments between the two pH conditions.

## Introduction

The existence of transparent exopolymer particles (TEP) was demonstrated by [1] using a polysaccharides-specific staining technique applying alcian blue. Defined as particles, TEP are gels spontaneously formed from polymeric dissolved organic matter (DOM), mainly composed of acid polysaccharides. TEP-precursors, colloidal and free fibril polymers, are produced by microorganisms, mainly by phytoplankton [2].Through their chemical composition (rich in carbon and trace elements) and high stickiness, TEP affect the food web structure and the carbon cycle in the ocean. In addition to be a source of carbon for bacteria, TEP formation is also an abiotic pathway to convert dissolved organic carbon (DOC) to particulate organic carbon (POC) *via* aggregation processes [3]. In the deep ocean, the evident correlation between fluxes of POC and minerals (e.g. clays, calcium carbonate, silica) led to the ballast hypothesis [4]. The accumulation of those so-called ‘mineral ballasts’ on organic aggregates was assumed to enhance the flux of POC by providing physical protection from degradation. An alternative explanation proposed by [5] is that the flux of POC controls the flux of the non-sinking mineral particles, due to the stickiness property of the organic material that allows the formation of large and fast-sinking particles. Since TEP are a matrix of organic aggregates, their formation would be an intermediary pool for carbon sequestration *via* sedimentation and an important component of carbon export to the deep ocean [6–8].

The work of [9] emphasized the importance of lithogenic particles as an important carrier phase of organic carbon export to the seafloor, suggesting that their exclusion from biogeochemical models leads to an underestimation of 16–51% of the POC flux to the deep ocean. Lithogenic particles are the second most important carrier phase for organic material export. They can enhance the export of fresh organic material by a shift in the formation and density of marine aggregates [10]. In addition to riverine derived lithogenic material, atmospheric dust deposition plays an important role in the export of POC. This is, for instance, the case in the dust-rich ocean regions off Northwestern Africa [11]. As suggested by [12], dust particles act as a catalyst for particle aggregation. This physical process would even have a more important effect on carbon export than the enhancement of POC production and export due to dust-mediated fertilization (i.e. nutrient release). In the Mediterranean western (MW) basin, several studies showed the enhancement of carbon export due to this aggregation process following a dust event [13–15]. The mechanisms involved included the aggregation of Saharan dust and dissolved organic matter (DOM) present in surface seawater. Dust addition experiments performed in filtered seawater by [14] suggested that the observed variability of abiotic aggregation depended on the *in situ* biogeochemical conditions at the time when filtered seawater was collected. In other words, the nature, concentration and age of dissolved organic matter (DOM) would control the formation of TEP, leading to seasonal variability in the export of POC mediated by the deposition of lithogenic particles.

The future evolution of organic carbon export to the deep ocean under anthropogenic pressure needs to be investigated in order to evaluate potential positive or negative feedbacks on atmospheric CO_2_ concentrations, and therefore on climate. As such, modifications of aggregation processes driven by TEP formation have the potential to affect carbon export. Although molecular processes controlling abiotic TEP formation are not yet well understood, it is known that the chemical and physical bonds (e.g. hydrogens bonds) involving the assembly of TEP-precursors are pH-dependent [16,17]. Therefore, an alteration of TEP formation under lowered pH conditions could be expected. Currently, the impact of ocean acidification on the formation and stickiness of TEP remains equivocal, and no changes in organic carbon export due to a shift in TEP dynamics have been postulated [18]. Furthermore, the affinity between polysaccharides, which are major TEP components, and mineral particles also depends on pH [8,19,20].There is therefore a great need to evaluate the effect of ocean acidification on the interaction between organic and lithogenic particles.

Our study was focused on the Mediterranean Sea, as it receives among the highest dust fluxes in the world ocean [21,22]. In the western Mediterranean region, mineral dust is mainly transported from the Sahara in the form of pulses [21,23–27]. The work of [28] reported annual mineral dust deposition fluxes ranging between 4 and 26.2 g m^−2^ (average 12.5 g m^−2^) between 1984 and 1994, with events larger than 0.5–1 g m^−2^ driving these annual fluxes and thus the interannual variability. The same orders of magnitude were found in more recent observations reported in [13], with an average annual dust flux over four years (2003–2007) of 11.4 g m^−2^ yr^−1^, mainly controlled by wet deposition. Smaller deposition rates have been reported between 2011 and 2013 in the Western Mediterranean Sea [27]. Over the past decade, the strongest deposition events reported in the Northwestern Mediterranean Sea (i.e ~22.2 g m^−2^) were observed at Cap Ferrat, French Riviera, on 26 February 2004, corresponding to 88% of the annual dust deposition that year [29], and in Corsica (21.9 g m^−2^ corresponding to 80% of the annual dust deposition that year) [30]. By measuring simultaneously atmospheric deposition and organic matter export in sediment traps in the Northwestern Mediterranean Sea during four years, [13] observed that the strongest POC fluxes were concomitant with large lithogenic export flux, originating from Saharan deposition events or from exceptional river floods. These associated organic export fluxes were defined as “lithogenic events” as they were, at least partly, attributed to organic-mineral aggregation inducing a ballast effect [13].

Another feature of the Mediterranean Sea is that it is considered as an oligotrophic basin with a west-east decreasing gradient in chlorophyll distribution. Similarly to the North Atlantic, in the more productive Northwestern Mediterranean Sea, a bloom is generally observed in late winter-early spring [31]. According to a nine-year time series at DYFAMED (43°25’ N, 7°52’ E), there is a well-defined seasonal succession of phytoplankton groups in this region which is related to variable hydrological conditions and nutrient concentrations. A shift of dominance from diatoms in winter to cyanobacteria during the stratified period is usually observed [32], leading to a clear seasonal variation of DOM concentrations [33]. The Northwestern Mediterranean Sea is thus an interesting area to conduct experiments under contrasted environmental conditions, with the objective to investigate the relationship between abiotic TEP formation following dust deposition and initial trophic conditions. The western Mediterranean basin is also an interesting area to perform ocean acidification perturbation experiments as it appears particularly impacted by this process. Indeed, a surface seawater pH decline of 0.15 pH units as compared to ~ 0.1 in world ocean has been estimated in this area since the beginning of the industrial era [34].

The main objective of the present study was to follow the effects of dust deposition, on organic matter dynamics and carbon export under present and future partial pressures of CO_2_ (*p*CO_2_) conditions. Thus, both natural (dust deposition) and anthropogenic (ocean acidification) forcing on TEP dynamics were investigated by means of three distinct experiments conducted in the Mediterranean western basin, at three seasons characterized by different initial trophic conditions.

## Material and methods

### Experimental setup and sampling schedule

**Fig 1:**
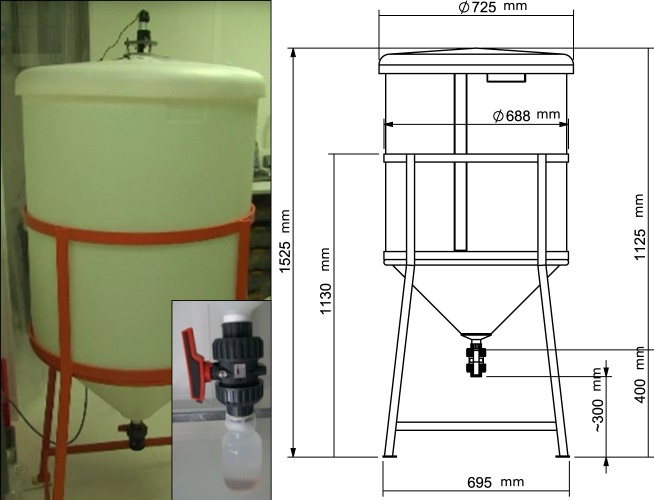
Picture and scheme (with dimensions in mm) of an experimental tank (minicosm).

Two minicosms made out of high-density polyethylene (HDPE) material (height: 1.09 m, diameter: 0.68 m, surface area: 0.36 m^2^ and volume: 0.31 m^3^, equipped with a sediment trap at their bottom) were installed inside a clean room in the dark and at constant temperature. At the base of each tank, a polyethylene (PE) bottle collecting the exported material from above, was screwed with a polyvinyl chloride (PVC) valve that remained open during the duration of the whole experiments (Fig 1). A weak turbulence was generated by a PVC blade, previously cleaned following trace-metal clean procedures and rotating at 9 rpm (rotations per minute).

Before each experiment, the minicosms were cleaned according to the protocol described by [14]. Briefly, they were first washed by a surfactant (DECON ® Neutracon ™), then they were kept with a 10% solution of hydrochloric acid (HCl) for one week, and finally with a 1% HCl Merck ™ Suprapur ® solution for another week. Between each step, minicosms were abundantly rinsed with ultrapure water. The final rinse was performed with the freshly collected filtered seawater before filling the minicosms.

A total of three experiments were conducted at three different seasons: during two ‘no bloom’ periods at the start of the stratification period (CHIPIE 1 initiated on May 21^th^ 2013) and at the end of the stratification period (CHIPIE 2 initiated on September 30^th^ 2013) as well as during the winter phytoplankton bloom (CHIPIE 3 initiated on January 28^th^ 2014). For each experiment, the two minicosms were filled with filtered seawater as described in [14]. Briefly, 600 L of seawater were collected at 5-m depth at the entrance of the Bay of Villefranche (France) using three trace metal clean Teflon pumps connected to polyethylene tubes. Inline filtration was performed using a 0.2 μm cartridge (Sartorius Sartrobran-P-capsule with a 0.45 μm prefilter and a 0.2 μm final filter). Trace-metal clean high density polyethylene (HDPE) containers (25 L) were filled with this filtered seawater and were kept in the dark until the transfer of seawater to the minicosms installed inside the clean room (less than 4 h for the entire process). Additionally, a subset of unfiltered seawater was sampled onboard and was used for the measurements of initial *in situ* conditions regarding TEP concentrations and heterotrophic prokaryote abundances. Pigment concentrations as measured by high-performance liquid chromatography (HPLC) from seawater samples collected weekly at the observation site “point B” in the Bay of Villefranche (43°41.1' N, 7°18.9' E; http://oov.obs-vlfr.fr/fr/observations.html) were used as biomarkers of several phytoplankton taxa. No specific permissions were required for this location because it is a public area to which we have full access due to its proximity to our laboratory. In addition, these studies did not involve endangered or protected species.

Filtered seawater was kept inside the minicosms for one night. The next morning, in one minicosm, referred to as “A” (acidified), *p*CO_2_ was increased to 1,250 μatm, slightly above the value projected for 2100 following scenario RCP8.5 [35] through addition of CO_2_-saturated seawater previously filtered onto 0.2 μm. In the second minicosm, *p*CO_2_ conditions were not modified, referred to as “NA” (non-acidified). A Saharan dust event (flux = 10 g m^-2^) was then simulated at the surface of both minicosms, mimicking a typical strong dust deposition event in the Northwestern Mediterranean Sea ([36], references therein). The methodology to obtain a dust analog is fully described in [30]. Briefly, the < 20 μm fraction of soil was collected in Southern Tunisia (33°25.38' N, 9°02.08' E) which appears to be a major source of dust deposition in the northwestern Mediterranean basin. The particle size distribution showed that 99% of particles had a size smaller than 0.1 μm, and that particles were mostly made of quartz (40%), calcite (30%) and clay (25%). In order to mimic the atmospheric transport of aerosols, a chemical artificial aging of dust analog was performed by the simulation of cloud evapocondensation cycles [30]. This consists in mixing dust with synthetic cloud water composed of inorganic acids (H_2_SO_4_ and HNO_3_) and oxalic acid (H_2_C_2_O_4_). Then the suspension is spread on polystyrene tray and dried under clean air flux. The artificial dust addition corresponded to 3.6 g of this dust analog diluted into 2 L of ultrapure water that was sprayed at the surface of the minicosms.

A depth-integrated polypropylene sampler (0–0.5 m) was used to perform discrete samplings for TEP, dissolved inorganic carbon (*C*_T_), DOC, salinity and total alkalinity (*A*_T_). Samples for *A*_*T*_ and DOC were filtered onto acid-cleaned 0.2 μm polycarbonate filters and onto GF/F filters (pre-combusted at 400°C during 4 h) respectively, under a laminar flow bench. After filtration, samples for DOC were immediately frozen. Samples for *A*_*T*_ and *C*_T_ were poisoned with a saturated solution of mercuric chloride (HgCl_2_) and kept in the dark at room temperature. Samples for the enumeration of TEP were fixed with a 37% buffered formaldehyde solution (1% final concentration) and stored at 4°C pending analyses. Abundance of heterotrophic prokaryotes was determined by flow cytometry. Sub-samples seawater (2 mL) were immediately fixed in glutaraldhehyde (Sigma, 1% final concentration), flash frozen in liquid nitrogen and stored at −80°C pending analysis [37].

Two control samplings were performed before the simulation of dust deposition: “C1” refers to the sampling performed before the acidification step in both minicosms and “C2” refers to the sampling performed after the acidification step in minicosm “A”. After seeding, sampling of both minicosms “NA” and “A” was performed simultaneously after 1.5, 12.5, 14, 24, 48, 72 and 144 h during CHIPIE 1. It was slightly modified during CHIPIE 2 and 3: 1, 6, 12, 24, 48, 72, 96 and 144 h. At the end of each experiment, the particulate material that settled during the experiment was collected in the sediment traps: the bottom valve of the minicosms was closed and the bottles removed. Samples were desalted with ultrapure water and freeze-dried.

Temperature and pH_T_ (on the total scale) were continuously monitored using, respectively, thermometers (pt1000) and Metrohm glass electrodes calibrated daily using a TRIS buffer solution (salinity 38). Data were recorded every minute on a Consort D230 datalogger.

Note that performing minicosm experiments did not reflect a true response to dust storm event and high *p*CO_2_ conditions in real open ocean, but they allowed to investigate the interactions between organic matter and lithogenic particles in filtered seawater and their potential responses to a decrease in seawater pH.

### Analytical protocols

#### Carbonate chemistry

From discrete samples, triplicate measurements of *A*_T_ were performed potentiometrically using a Metrohm titrator (Titrando 888) and a glass electrode (Metrohm, ecotrode plus) and *A*_T_ was calculated as described in [38] *C*_T_ was determined on triplicate 1.2 mL subsamples using an inorganic carbon analyzer (AIRICA, Marianda, Kiel, Germany) coupled to an infrared gas analyzer (LI-COR 6262). Salinity was determined using an AUTOSAL salinometer. The carbonate chemistry was assessed using *C*_T_, *A*_T_, temperature and salinity using the R package seacarb [39].

#### Dissolved organic carbon

Duplicate samples for DOC measurements were unfrozen at room temperature, acidified with 18 μL of phosphoric acid (H_3_PO_4_; pH < 3), and analyzed in triplicate by high-temperature catalytic combustion on a Shimadzu TOC-VCPH analyzer with an ASI-V auto-sampler. All inorganic carbon and purgeable organic carbon was removed by a sparging process of acidified samples.

#### Heterotrophic prokaryote abundances

Heterotrophic prokaryote abundance was determined by flow cytometry according to [37]. Samples were diluted 2 -fold in autoclaved and 0.2 μm prefiltered TE buffer in order to avoid coincidence and then stained with (1:10000v/v) SYBR Green I (Molecular Probes). Samples were then incubated for 10 min in the dark, and analyzed with the FACSCalibur flow cytometer.

#### TEP abundance and size number distribution

A microscopy method, based on (2) and modified by [40], was used for the enumeration of TEP. Briefly, an aliquot of 40 mL from each minicosm were filtered onto 0.2 μm white Nuclepore filters (25 mm Ø). After filtration under constant and low pressure, materials retained on filters were stained with a solution of 0.06% acetic acid and 0.02% Alcian blue 8GX (previously filtered onto 0.2 μm before each experiment). Filters were mounted directly on a white slide (Cyto-Clear®) and TEP were observed by an Axiophot-Zeiss microscope coupled to a semi-automatic image-analysis system (Image Pro + 4.5 ^TM^ software). TEP were counted and sized at two successive magnifications (x200 and x400), and classified into 15 logarithmic size classes between 1 and 135 μm. TEP abundance ([TEP]) was given, in numbers of TEP mL^-1^ (# mL^-1^) [41]. The microscopy method used for estimating the abundance of TEP is a quantitative method although counting-related errors can vary from 3 to 20% [41]. A conservative error of 20% has been considered during this experiment, and standard deviations (SD) have been calculated accordingly.

In order to determine the size evolution of TEP during the experiments, the TEP number size distribution was calculated using a power law [42]:

$$\text{d}N/\text{d}\left(d\text{p}\right){=\text{k x dp}}^{\delta }$$

where d*N* is the number of particles per unit volume, with a diameter ranging from *d*p to [*d*p + d(*d*p)], and *d*p is the projected spherical encased diameter. The constant *k* depended on particle concentration and the spectral slope δ indicated the abundance of small to large particles. The TEP abundance belonging to a given size class was standardized by the width of this size class (# mL^-1^ μm^-1^).

The average diameter D_av_ (μm) was calculated as follows:

$${\text{D}}_{\text{av}}=\frac{\sum_{\text{i}}\left(\text{N}(d\text{pi})\times d\text{pi}\right)}{\sum_{\text{i}}\text{N}(d{\text{p}}_{\text{i}})}$$

where the symbol i is the different size classes over the 1–135 μm size range, *d*p_i_ (μm) and N(*d*p_i_) (# mL^-1^) are respectively the mid-point diameter and the TEP abundance of a given size class. ∑_i_ N(*d*p_i_) refers to the total TEP abundance.

TEP volume concentration (C_v_) given in ppm, was calculated as follows:

$${\text{C}}_{\text{v}}=\frac{\pi \times {d\text{pi}}^{3}}{6}\times \text{N}(d{\text{p}}_{\text{i}})\times 10^{-6}$$

#### Particulate material in sediment traps: Total mass, POC and lithogenic fraction

Particulate material fluxes were measured by weighing freeze-dried samples five times. The weight percentage of total carbon (% TC) in the exported material was measured with an Elementar Vario El analyzer on aliquots (15 mg) of freeze-dried sediment trap samples. A second aliquot (~ 20 mg) was acid digested (HNO_3_/HF at 150°C) [43] before analysis by inductively coupled plasma atomic emission spectroscopy (ICP-AES). Assuming that particulate inorganic carbon (PIC) was mainly associated to calcium carbonate (CaCO_3_), the measurement of calcium (% Ca) by ICP-AES allowed calculating the % of PIC (% Ca x 12/40, with 12 and 40 corresponding to molar masses of carbon (C) and calcium (Ca), respectively). The weigh percentage of POC (% POC) was then determined by subtracting % PIC from % TC. Results are expressed in mmol POC collected in each sediment trap. POC fluxes were then calculated considering the surface of the minicosms (0.36 m^2^) and daily fluxes were calculated considering the duration of the experiments (6 days). Using aluminum (Al) concentrations as a proxy for dust concentrations (Al = 4.12 ± 0.39% in the dust analog), the measurement of Al content (%) in the exported material by ICP-AES allowed to estimate the percentage of lithogenic particles recovered in the sediment trap and the fluxes of lithogenic particles over the course of the experiments.

## Results

### Initial *in situ* conditions

At the time of water collection for CHIPIE 1 and 2, pigments indicated that *in situ* plankton communities were mostly composed of small species (prymnesiophyceae and cyanobacteria; data not shown). At the time of CHIPIE 3 experiment, the *in situ* assemblage was characterized by a higher abundance of dinoflagellates and diatoms, in agreement with a higher autotrophic biomass during this winter-bloom ([Chl*a*] = 0.97 μg L^-1^; Table 1). *In situ* heterotrophic abundance (9.14 x 10^5^ cell mL^-1^) and TEP concentrations ([TEP]; 2426 # mL^-1^) were maximal during the spring experiment (CHIPIE 1). The three experiments were contrasted in terms of [DOC] with the lowest value observed during the winter phytoplankton bloom (CHIPIE 3).

**Table 1:** Comparison between (1) *in situ* unfiltered (noted ‘*in situ’*), (2) filtered seawater sampled inside the minicosms before the acidification step (noted “C1”) and (3) filtered seawater sampled inside the minicosms after acidification (noted “C2”).

	[Chl*a*]	[HP]					[TEP] ± SD					[DOC] ± SD				
	μg L^-1^	x10^5^ cell mL^-1^					# mL^-1^					μmol L^-1^				
	*in situ*	*in situ*	“C1”		“C2”		*In situ*	“C1”		“C2”		*In situ*	“C1”		“C2”	
			NA	“A”	“NA”	“A”		“NA”	“A”	“NA”	“A”		“NA”	“A”	“NA”	“A”
CHIPIE 1	0.04^a^	9.1	2.0	1.9	2.0	2.0	2426 ± 485	67 ± 13	20 ± 4	65 ±13	10 ± 2	ND	98 ± 1	ND	78 ± 1	ND
CHIPIE 2	0.15^a^^b^	6.5	0.4	0.4	0.5	0.3	1424± 285	67 ± 13	58 ± 12	35 ± 7	35 ± 7	74 ± 4	78 ± 0	80 ± 4	80 ± 2	82 ± 2
CHIPIE 3	0.97^a^	8.5^a^	2.5	2.5	2.6	2.6	1260± 252	129 ± 26	240 ± 48	106 ± 21	81 ± 16	66 ± 9	58 ± 8	54 ± 2	60 ± 2	64 ± 1

Heterotrophic prokaryote abundances ([HP]), transparent exopolymer particle concentrations [TEP] and dissolved organic carbon concentrations ([DOC]) are shown. “NA” and “A” correspond to the non-acidified minicosm and acidified minicosm, respectively. In addition, concentrations of chlorophyll *a* [Chl*a*] in seawater collected at the three distinct seasons are presented. Note that the time delay between C1 and C2 is ~ 3 h. ND refers to as not determined. [TEP] and [DOC] standard deviations (SD) are also shown. For [DOC], SD were calculated based on duplicate analyses and for [TEP] a conservative maximal error of 20% on TEP microscopic enumeration has been considered, see text for details.

^a^data from SOMLIT (http://somlit.epoc.u-bordeaux1.fr/fr/).

^b^[Chl*a*] linearly interpolated between September 24^th^, 2013 ([Chl*a*] = 0.12 μg L^-1^) and October 8^th^, 2013 ([Chl*a*] = 0.18 μg L^-1^).

### Experimental conditions

Comparison between *in situ* unfiltered and filtered seawater showed a decrease, after filtration, of heterotrophic prokaryote abundances by 70 to 95% and a decrease of [TEP] by 85 to 98%, for the three experiments. Bacterial abundance remained low and stable between the start of each experiment (C1) and the first twelve hours after seeding (T12): 2.05 ± 0.10 (CHIPIE 1), 0.32 ± 0.09 (CHIPIE 2) and 2.54 ± 0.09 x 10^5^ cell mL^-1^ (CHIPIE 3). Then, a moderate bacterial growth was observed over the rest of each experiment, leading to a maximal bacterial abundance of 12.25 ± 0.35 (CHIPIE 1 at T144), 3.84 ± 0.62 (CHIPIE 2 at T72) and 12.40 ± 0.57 x 10^5^ cell mL^-1^ (CHIPIE 3 at T144; S1 Fig).

The filtration allowed removing all autotrophs including small species such as cyanobacteria as no phytoplankton were depicted during the whole duration of the experiments (daily sampling and measurement with a FACSCalibur flow cytometer, bivariate scatterplots green fluorescence (FL1-H) versus red fluorescence (FL3-H)–data not shown).

### Temperature, pH_T_, dissolved inorganic carbon and alkalinity

Average seawater temperature (± standard deviation; SD) was 20.3 ± 0.3, 22.3 ± 0.3 and 19.4 ± 0.4°C over the six experimental days of CHIPIE 1, 2 and 3, respectively. pH_T_ in “NA” *vs* “A” were, on average (± SD), 8.0 ± 0.1 *vs* 7.6 ± 0.1 (CHIPIE 1), 8.0 ± 0.0 *vs* 7.6 ± 0.1 (CHIPIE 2) and 8.1 ± 0.0 *vs* 7.7 ± 0.1 (CHIPIE 3)_._ *C*_T_ averaged (± SD) 2292 ± 6 *vs* 2489 ± 4 (CHIPIE 1), 2251 ± 6 *vs* 2467 ± 32 (CHIPIE 2), and 2265 ± 6 *vs* 2488 ± 35 μmol kg^-1^ (CHIPIE 3). *A*_T_ remained constant in both “NA” and “A”, and averaged (± SD) 2549 ± 4, 2535 ± 13 and 2537 ± 6 μmol kg^-1^ during CHIPIE 1, 2 and 3, respectively.

### Dissolved organic carbon, carbon export and lithogenic flux

Along the course of the experiments (144 h), [DOC] remained constant in both treatments averaging (± SD) 96 ± 14 (CHIPIE 1), 90 ± 7 (CHIPIE 2) and 65 ± 5 μmol L^-1^ (CHIPIE 3) (S2 Fig). The exported POC (in mmol C) collected in sediments traps after 144 h in “NA” *vs* “A” were of 1.1 *vs* 1.2 (CHIPIE 1), 2.7 *vs* 2.6 (CHIPIE 2) and 2.0 *vs* 2.5 (CHIPIE 3). This corresponded to fluxes over six days in the non-acidified minicosm of 38.2 (CHIPIE1), 90.1 (CHIPIE 2) and 67.5 mg m^-2^ (CHIPIE 3; Table 2). Fluxes were very similar in the acidified minicosm during CHIPIE 1 and 2 (38.7 and 87.1 mg m^-2^, respectively) but slightly higher during CHIPIE 3 (81.7 mg m^-2^).

**Table 2:** Abiotic carbon export following an artificial dust deposition.

	POC to dry weight ratio		Stock		Stock		Flux		Flux		Flux	
	(%)		mmol C		mg C		mmol m^-2^		mg m^-2^		mg m^-2^ d^-1^	
	“NA”	“A”	“NA”	“A”	“NA”	“A”	“NA”	“A”	“NA”	“A”	“NA”	“A”
CHIPIE 1	1.6	1.6	1.1	1.2	13.8	13.9	3.2	3.2	38.2	38.7	6.4	6.4
CHIPIE 2	1.5	1.5	2.7	2.6	32.4	31.3	7.5	7.3	90.1	87.1	15.0	14.5
CHIPIE 3	1.3	1.6	2.0	2.5	24.3	29.4	5.6	6.8	67.5	81.7	11.3	13.6

The weight percentage of particulate organic carbon (POC; %) measured in the sediment traps collected at the end of each CHIPIE experiment in both minicosms, the stock (mmol and mg of carbon), flux of POC (mmol C m^-2^ and mg C m^-2^) exported over six experimental days, and the average flux of POC per day (mg m^**-**2^ d^-1^).

Whatever the experiment, the organic carbon content of the particulate material, collected in sediment traps at the end of the experiments, was identical, with an average POC to dry weight ratio of 1.5 ± 0.1% for all experiments (Table 2). Based on Al measurements, the percentage of dust recovered in the sediment traps six days after seeding, ranged from 31.0 to 31.6% (CHIPIE 1), from 73.1 to 73.7% (CHIPIE 2), and from 54.6 to 61.9% (CHIPIE 3; Table 3). Finally, over six days of all experiments, the lithogenic flux varied from 3,100 to 7,370 mg m^-2^ (Table 3).

**Table 3:** Mass budget in sediments traps and determination of lithogenic fluxes.

	Al content (%)		Al mass (mg)		% dust exported		Flux (mg m^-2^)	
	“NA”	“A”	“NA”	“A”	“NA”	“A”	“NA”	“A”
CHIPIE 1	5.3	5.4	46.0	46.8	31.0	31.6	3,100	3,160
CHIPIE 2	4.9	5.1	108.4	109.2	73.1	73.7	7,310	7,370
CHIPIE 3	4.4	4.9	81.1	91.8	54.6	61.9	5,460	6,190

The weight percentage (%) and the mass (mg) of aluminium (Al; %) measured in the sediment traps collected at the end of each CHIPIE experiment in both minicosms. Using Al as a proxy of lithogenic particles, the percentage of dust recovered in the sediment trap (% dust exported) and the flux of lithogenic particles were estimated.

### TEP abundance

**Fig 2:**
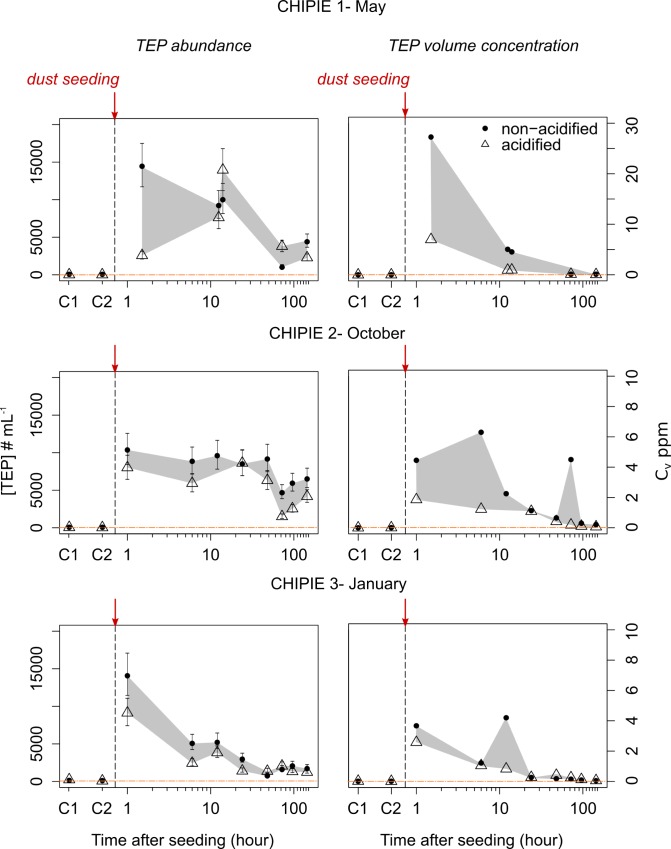
Abundance ([TEP] in # mL^-1^) and volume concentration of transparent exopolymer particles (C_v_ in ppm) along the course of the three CHIPIE experiments in the two minicosms (non-acidified and acidified). “C1” and “C2” refer to samplings performed before and after acidification, respectively. The filled area represents the range of TEP abundance and volume concentration based on the results in the minicosm non-acidified (black circles) and acidified (empty triangle). The x-axis (in log-scale) represents the number of hours after the artificial dust deposition at the surface of both minicosms represented as a vertical dashed line. Error bars correspond to standard deviations estimated considering a conservative counting error of 20%.

In both minicosms, at sampling time “C2” (before seeding in both minicosms and after acidification in “A”), [TEP] remained low during CHIPIE 1, 2 and 3 (ranging from 10 to 106 # mL^-1^; Table 1). This represents less than 1% of maximal TEP abundance measured during the first hours after seeding. As shown in Fig 2 by considering a maximal counting error of ± 20%, TEP abundance in “NA” ([TEP]_NA_) was maximal after seeding: 14,449 ± 2,890 (at T1.5), 10,364 ± 2,073 (at T1) and 14,070 ± 2,814 # mL^-1^ (at T1) during CHIPIE 1, 2 and 3, respectively. [TEP]_NA_ then decreased to reach 4,401 ± 880 (CHIPIE 1) and 1,706 ± 341 (CHIPIE 3) # mL^-1^ at T144. During CHIPIE 2, [TEP]_NA_ remained stable until T48 (9,159 ± 1,832 # mL^-1^), dropped at T72 and reached a minimum of 6,508 ± 1,302 # mL^-1^ at T144.

During CHIPIE 2 and 3, the TEP abundance in “A” ([TEP]_A_) followed the same trend than [TEP]_NA_ (Fig 2) One hour after seeding, [TEP]_A_ reached 8,011 ± 1,602 (CHIPIE 2) and 9,125 ± 1,825 # mL^-1^ (CHIPIE 3), and then decreased to 4,186 ± 837 and 1,238 ± 248 # mL^-1^ at the end (T144) of CHIPIE 2 and 3, respectively.

### TEP size distribution

**Fig 3:**
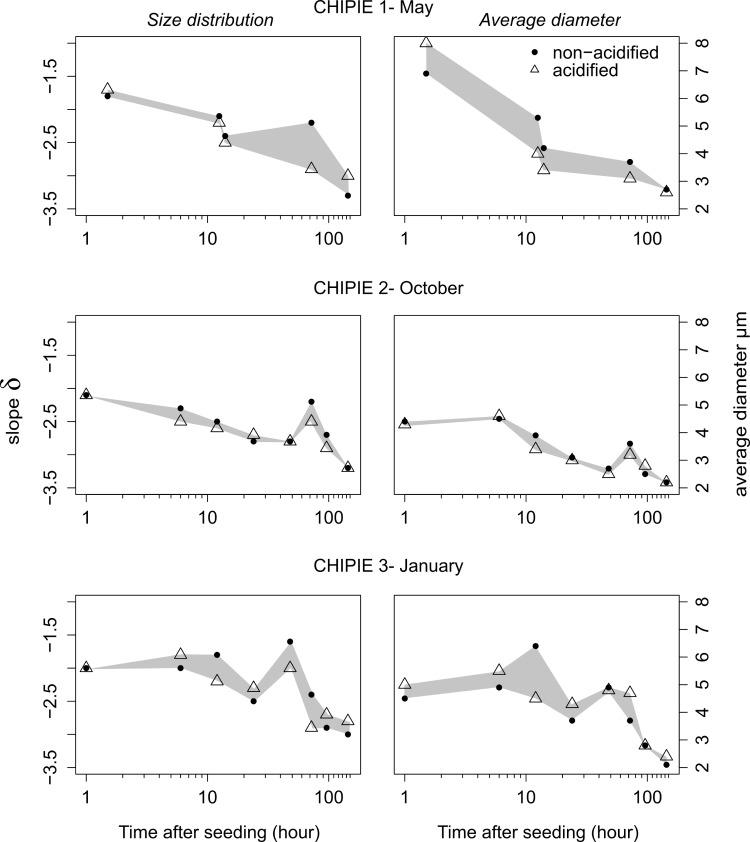
Evolution of the spectral slope (δ) of the size distribution of transparent exopolymer particles (TEP; left panels) and the average diameter of TEP (right panels) in both minicosms after seeding for all CHIPIE experiments. When δ decreases, the relative proportion (in number) of small particles increases. Circles and triangles represent data from the non-acidified and acidified minicosm, respectively.

Just after seeding, the spectral slope (δ) was, in both minicosms, -1.7 [CHIPIE 1 (T1.5)], -2.1 [CHIPIE 2 (T1)] and -2 [CHIPIE 3 (T1)]. The relative abundance of small particles increased during the three experiments independently of *p*CO_2_ conditions to reach -3.3 (CHIPIE 1), -3.2 (CHIPIE 2) and -3.1 (CHIPIE 3) in “NA”, and -3.0 (CHIPIE 1), -3.2 (CHIPIE 2) and -2.8 (CHIPIE 3) in “A” (Fig 3). Additionally, the average diameter, estimated in both minicosms, decreased over all CHIPIE experiments (Fig 3). Just ~ 1 hour after seeding, the average diameter of TEP abiotically formed in “NA” *vs”*A” was 6.9 *vs* 8.0 (CHIPIE 1), 4.4 *vs* 4.3 (CHIPIE 2), and 4.5 *vs* 5.0 μm (CHIPIE 3). At the end of the experiments (T144), the average diameter was around of 2.4 ± 0.2 μm. Note that the peak of δ observed in CHIPIE 2 and 3 at T72 and T48, respectively, was concomitant with the increase in the average diameter, followed then by a decrease in these two parameters.

### TEP volume concentration

The estimation of TEP volume concentration (C_v_; ppm) allowed to take in consideration the number and size of TEP to assess TEP dynamic over experiments. For CHIPIE 1, the high C_v_ of 27.3 ppm observed at T1.5 in “NA” highlighted the larger diameter of the produced TEP (Fig 3). As with TEP abundance, C_v_ decreased until the end of each experiment after reaching the maximal value during the first hours after seeding. It must be stressed that the peak of C_v_ observed at T72 in “NA” during CHIPIE 2 was concomitant with a decrease in [TEP]_NA_ and an increase in TEP size (Figs 2 and 3).

## Discussion

It is well known that TEP can be formed *via* either biotic or abiotic processes [8]. Abiotic formation can be enhanced by bubble adsorption, surface coagulation, and turbulent and laminar shear [7,44,45]. In addition to these physical processes, TEP formation could be enhanced by adsorption of TEP precursors onto particles because of strong interactions between polysaccharides and surface of mineral particles [8,16,19,20]. Our experiments were performed using seawater from which almost all TEP particles have been initially removed. While two control samplings performed before the simulation of dust deposition (~3 h time laps) showed no TEP formation, the addition of dust clearly accelerated the kinetics of TEP formation. The absence of cyanobacteria cells and bacterial growth during the first hours after seeding indicated that TEP formation was related to abiotic processes between the organic precursors, initially present in collected seawater, and dust. Our fine sampling strategy allowed evidencing a rapid abiotic formation (≤ 1 h after seeding) of predominantly large TEP. Based on all CHIPIE experiments, TEP abundances measured one hour after seeding were 5 to 10 times larger than *in situ* concentrations.

**Fig 4:**
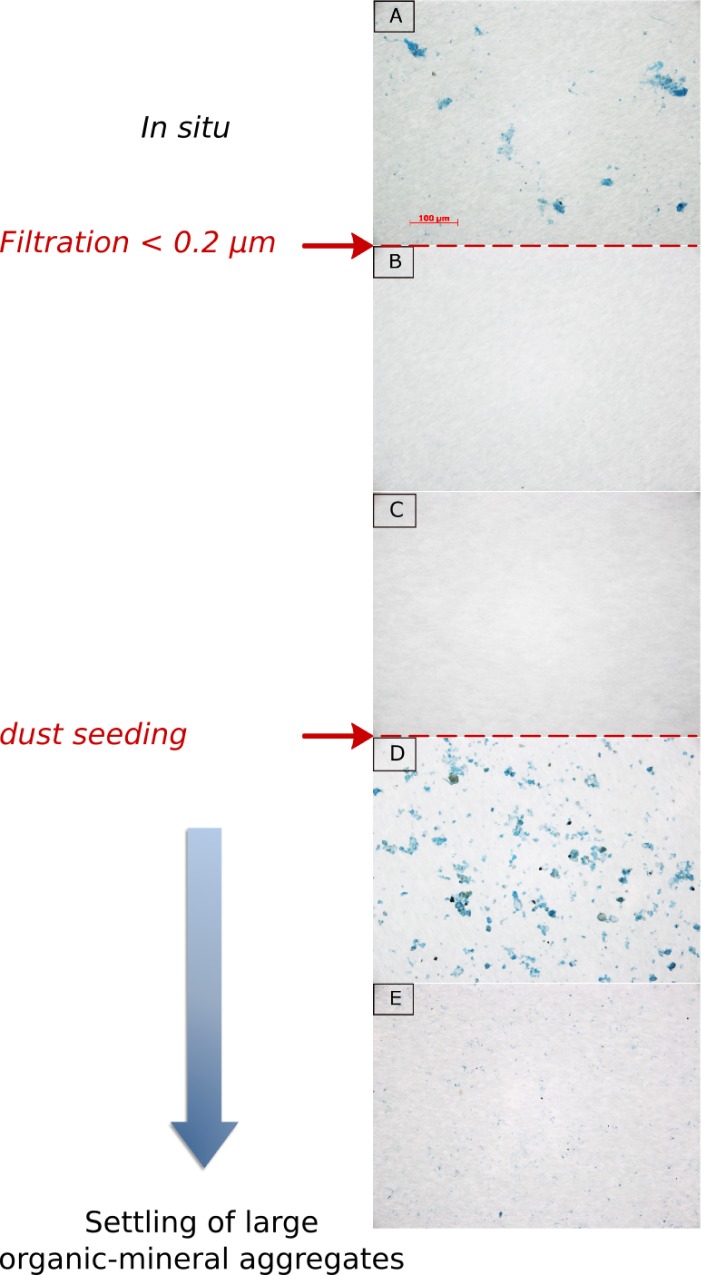
Microscopic image (x 200) of transparent exopolymer particles (TEP) stained with alcian blue. In seawater collected in the Bay of Villefranche (unfiltered) on May 21^st^ 2013 (A), after filtration “C1” (B) and “C2” (C), 1 hour after dust deposition (D) and at the completion of this CHIPIE 1 experiment (six days after dust addition; E).

The evolution of TEP size distribution followed the same trend over all experiments with the average diameter and the relative proportion of large TEP decreasing over time after the initial increases. The peak of the spectral slope and diameter observed during CHIPIE 2 and 3 (72 and 48 h after seeding respectively) demonstrates an aggregation process of TEP. This process was well visible during CHIPIE 2 with the increase in TEP volume concentration at 72 h while TEP abundance dropped at the same time. Due to their stickiness property, TEP can aggregate themselves but also act as a glue for the particulate material. [15] showed, in a natural planktonic assemblage, that a significant fraction of the POC flux (36–50%) was associated with lithogenic particles *via* aggregation processes after a Saharan dust deposition event. In the absence of photoxidation and the negligible effect of bacterial growth on the stock of organic carbon (from the estimation of carbon consumption) during our experiments, the decline in abundance and/or in size of TEP observed after reaching the maximal TEP production likely illustrates the settling of the formed aggregates between the organic matter and dust (Fig 4). While TEP abundance declined along the course of CHIPIE 3, it remained quite constant for two days after seeding in CHIPIE 2. One possible explanation is that, during this experiment, TEP concentrations were not yet in equilibrium with their precursors and their production continued during the settling of large aggregates. This experiment showed that, under oligotrophic conditions ([NO_3_^-^] = 0.05 μM; [PO_4_^3-^] = 3.4 nM; source: SOMLIT, http://somlit.epoc.u-bordeaux1.fr/fr/), TEP abiotically formed following a dust event can remain in suspension for a longer time compared to what was observed for winter bloom conditions (CHIPIE 3).

**Fig 5:**
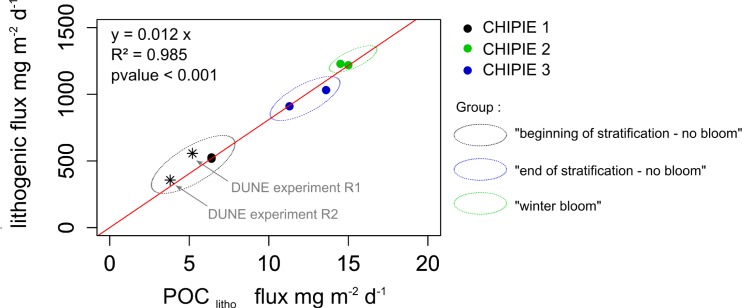
Relationship between the integrated lithogenic flux (mg m^-2^ d^-1^) and the particulate organic carbon (POC) flux (mg m^-2^ d^-1^) associated to the lithogenic ballasting (POC_litho_) over 6 days of each experiment. Based on the results of the present study (black circles), a correlation equation was established with a slope of 0.012 (pvalue < 0.001, r^2^ = 0.985). Stars represent data from DUNE experiments R1 and R2 [15,46].

With respect to the fate of dust in the water column, not all the introduced dust was recovered six days after seeding and important differences in the amount recovered (as a percentage of the mass initially added) were observed between the experiments: from 31% (CHIPIE 1) up to ~74% (CHIPIE 2). During the DUNE experiments (*in situ* mesocosms deployed in the same area of the Mediterranean Sea in summer), a proportion of dust particles (same analog as during our experiments) with a slow-sinking velocity remained in suspension and escaped export to the sediment traps [15]. At the end of these mesocosms experiments, between 32 and 70% of the added dust was recovered in these sediment traps, directly associated with a difference in the export of POC [46]. During our experiments, fluxes of dust (Flux litho) and POC associated to lithogenic ballasting (Flux POC_litho_; mg m^-2^ d^-1^) were proportional (Fig 5):

$${\text{Flux POC}}_{\text{litho}}=0.012\times \text{Flux litho}$$

During the two *in situ* DUNE experiments [15,46], lithogenic fluxes were estimated to 3,342 and 2,139 mg m^-2^ (DUNE experiment R1 and R2 respectively) over six days after the first artificial dust deposition. By normalizing the POC flux measured in dust-seeded mesocosm to the integrated primary production, POC_litho_ fluxes of 31 (DUNE R1) and 23 mg m^-2^ (DUNE R2) were estimated [15]. A similar proportion between POC_litho_ and lithogenic fluxes was observed for the DUNE experiments and the CHIPIE experiments (Fig 5). This would allow using this relationship to extrapolate POC_litho_ fluxes induced by dust deposition events in this region and quantify their contribution to the flux of POC at different time scales. For instance, after an extreme Saharan dust event observed in February 2004 (22 g m^-2^), a POC_litho_ flux of 0.27 g m^-2^ within the following month could be estimated. This would correspond to 34% of the total measured POC export (0.8 g m^-2^) [13] after this deposition event, suggesting that the remaining fraction would likely be a combination of biomineral (CaCO_3_, opal) ballasting and export from enhanced primary production due to nutrient release. This shows the relevance of the impact of dust deposition on the export of POC_litho_ at the event timescale. Indeed, our daily contribution of POC_litho_ to total POC flux after the simulation of a strong dust deposition ranged from 6.4 to 15 mg m^-2^ d^-1^, which is of the same order of magnitude of the daily total POC flux per day (6.8 mg m^-2^ d^-1^) [47] in the Northwestern Mediterranean Sea. Nevertheless, at the annual scale, with an average dust flux of 11.4 g m^-2^ yr^-1^ [13] and a POC flux of 2.4 g m^-2^ yr^-1^ at the DYFAMED station (1988–2005 at 200 m depth) [47], POC_litho_ flux would only correspond to ~ 6% of the annual carbon export.

According to our experimental protocol, the POC exported originated from the conversion of DOC to POC mediated by TEP formation/aggregation was triggered by the introduction of dust. The POC collected after six days (T144h) represented a loss of 4, 11 and 10% of the initial DOC stock during CHIPIE 1, 2 and 3, respectively. Such small decreases in [DOC] fall within the analytical (triplicate measurements) and sampling (duplicates) errors (2 and 4–9% respectively), and could not be evidenced (S1 Fig). Considering that the single source of particulate organic carbon produced comes from the transfer of polysaccharides fraction, these values are in the range of the polysaccharides contribution (5–40%) to DOC concentration in surface waters of the ocean [48].

During our minicosm experiments, filtered seawater collected at three distinct seasons, received the same initial dust flux (10 g m^-2^). The linear relationship observed between POC_litho_ and lithogenic fluxes in the traps suggests that the organic matter, which acts as “glue”, likely controls the flux of lithogenic particles, as assumed by [5,49]. Depending on the initial *in situ* conditions, the export of particulate matter is more or less important although the saturating capacity of organic matter for lithogenic particles, as defined in [49], remains constant at 98.4–98.7 weight percentage mineral (i.e., a POC to dry weight ratio of 1.3 to 1.6%, Table 2). It has been suggested that the production of TEP is higher under nutrient limitation, with TEP being stickier and richer in carbon [42,50–52]. The nature of TEP-precursors reflecting the difference in quality of dissolved organic matter exuded from different phytoplankton groups [8], the role of TEP acting as a “glue” would be more or less efficient for the formation of sinking organic-dust aggregates. This should be reflected by our dataset since our three distinct experiments were conducted in contrasted conditions: CHIPIE 1 at the beginning of stratification under nutrient stress soon after the production of fresh DOM during the spring bloom; CHIPIE 2: at the end of the stratification with an accumulation of DOM not freshly produced under oligotrophic conditions and CHIPIE 3: during the winter bloom when fresh DOM is produced. Yet, during our CHIPIE 1 experiment, abiotic POC fluxes, measured six days after seeding, were lower compared to those in CHIPIE 2 and 3. This could result from a difference in aggregate formation, as highlighted by the larger diameter of the produced TEP. Our limited dataset concerning the nature of the TEP precursors prevent to explore into more details this hypothesis. Indeed, the link between the nature of TEP precursors (i.e. surface active carbohydrates content), TEP stickiness and aggregates formation needs to be further investigated.

The objective of the present study was also to assess the effect of ocean acidification on TEP dynamics and carbon export after dust addition. Despite the fact that average TEP abundance, as counted ~ 1 h after seeding, was always lower under high *p*CO_2_ conditions for all experiments, no conclusions could be drawn based only on this single sampling. Indeed, when considering the complete dataset for each experiment and counting errors of ± 20%, no clear effect of ocean acidification on the variation of TEP abundances as a function of time could be evidenced between non-acidified and acidified conditions (Fig 2). Similarly, TEP size distribution was also unlikely impacted by elevated *p*CO_2_ (Fig 3). This stands in contrast to what was observed in batch cultures of coccolithophores by [53], where an increase in the contribution of smaller size TEP under elevated *p*CO_2_ was shown. However, our results are in agreement with [54] who indicated that ocean acidification would not affect the abiotic formation of TEP and the equilibrium between TEP and their precursors. With respect to carbon export, we cannot conclude from our results on an effect of ocean acidification on the abiotically-mediated POC flux (Table 2). The formation of aggregates was likely independent of *p*CO_2_. This is in good agreement with laboratory experiment conducted by [55] on the effect of ocean acidification on the dynamics and characteristics of aggregates formed from marine detritus in the presence or absence of clay mineral illite.

## Conclusions

The high resolution sampling protocol considered during our experiments allowed highlighting the role of lithogenic particles on the dynamics of TEP formation following a dust deposition event. This work emphasizes the potential importance of DOM/dust interactions in rapid POC export according to contrasted *in situ* trophic conditions.

Saharan dust provoked the abiotic formation of TEP within the first hours after deposition at the surface of the minicosms allowing the formation of fast sinking organic-mineral aggregates and POC export. This export of “lithogenic POC” (POC_litho_) was variable depending on the season. Indeed, our data suggest that the efficiency of the “lithogenic carbon pump”, as defined by [15], is likely driven by the physicochemical characteristics of TEP-precursors that depend on the composition and physiological state of the phytoplankton community. Our results, combined with the ones of [15] obtained using *in situ* large mesocosms, showed a consistent linear relationship between the lithogenic mass flux and POC_litho_ (POC_litho_ flux = 1% of lithogenic flux). At the event timescale, the lithogenic carbon pump would be an important pathway of organic carbon export in region submitted to dust deposition with low productivity such as the Mediterranean Sea, and should be considered in biogeochemical models. Its parameterization needs further investigation, such as the link between the reactivity of organic matter and the physicochemical characterization of TEP-precursors depending on auto-heterotrophic assemblage.

With respect to the fate of this pump under high *p*CO_2_ conditions, no effect on TEP dynamics and POC flux could be evidenced in the present study. In order to assess the response of the dust-mediated POC flux to ocean acidification, future experimental efforts should be undertaken under natural (i.e. biotic) conditions. In addition, the assessment of the effect of temperature on the TEP formation stimulated after dust addition should be investigated. Indeed, a shift in the physico-chemical parameters of seawater such as temperature has the potential to modify the structure and the formation of TEP [17].

## Supporting information

S1 Fig Heterotrophic prokaryote abundance (cell mL^-1^) along the course of the three CHIPIE experiments in the two minicosms (non-acidified and acidified). (TIF)

S2 Fig Concentration of dissolved organic carbon ([DOC] in μmol L^-1^) along the course of the three CHIPIE experiments in the two minicosms (non-acidified and acidified). “C1” and “C2” refer to samplings performed before and after acidification, respectively. The x-axis (in log-scale) represents the number of hours after the artificial dust deposition at the surface of both minicosms represented as a vertical dashed line. The error bars correspond to the sum of the sampling and analytical errors for each point.
(TIF)
