# Supplementary figures and images for: Experimental evidence of formation of transparent exopolymer particles (TEP) and POC export provoked by dust addition under current and high pCO2 conditions
Source: PLoS One. 2017 Feb 17;12(2):e0171980. doi: 10.1371/journal.pone.0171980 (PMC5315277; doi:10.1371/journal.pone.0171980)

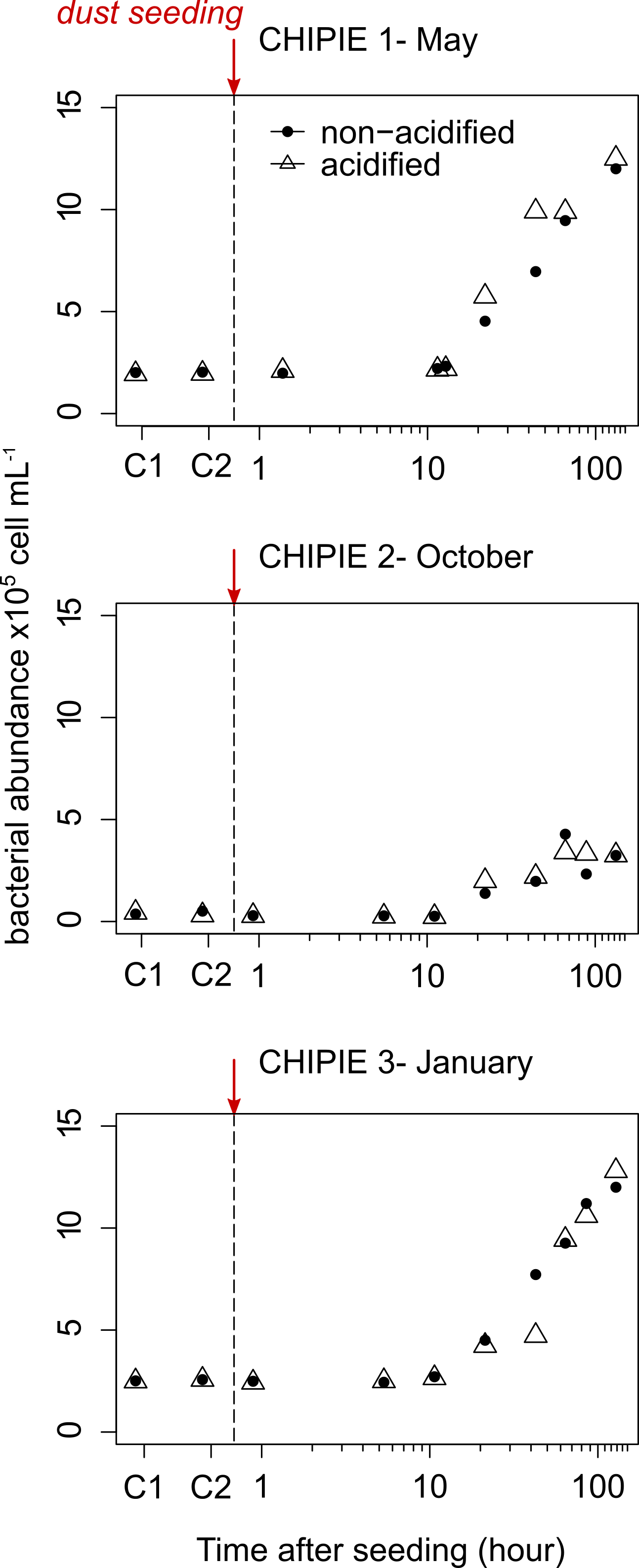

Supplement: S1 Fig — (TIF) [file pone.0171980.s001.tif]

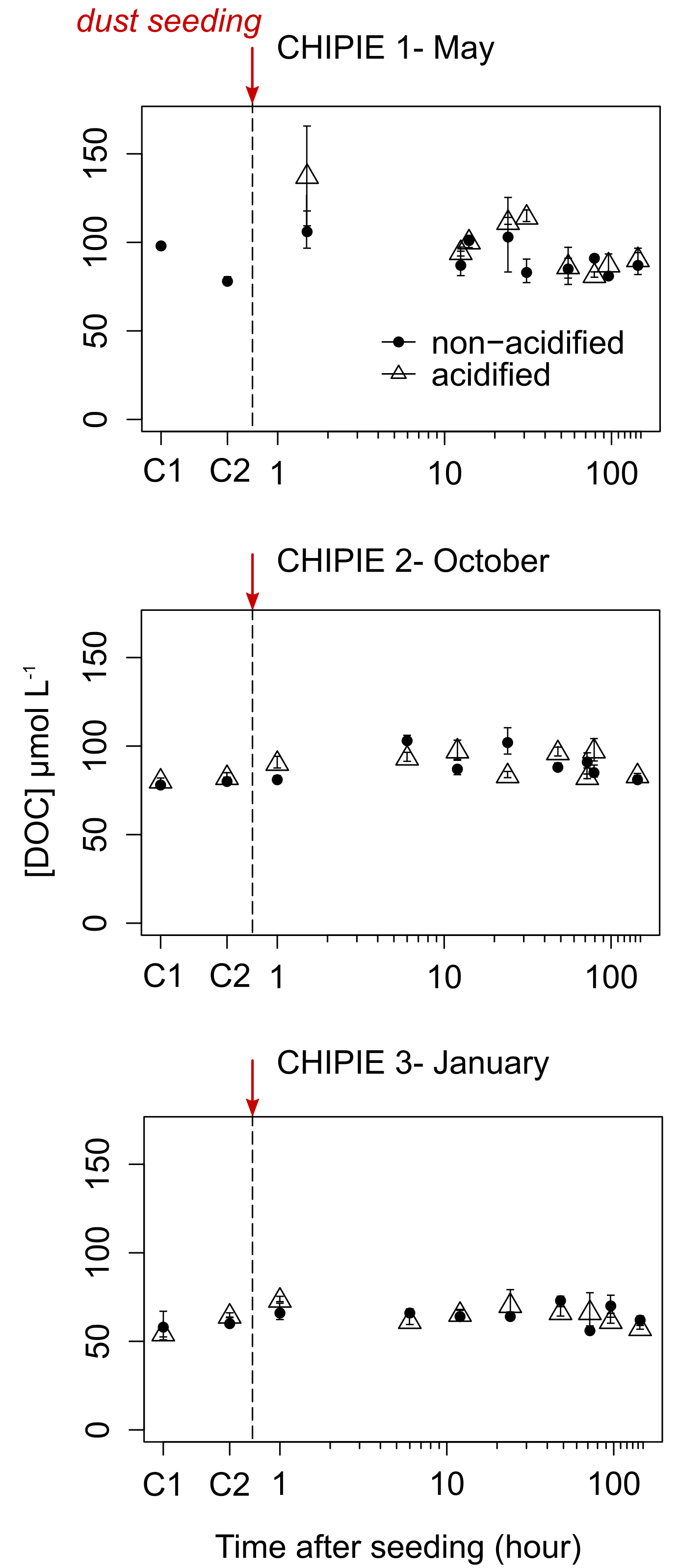

Supplement: S2 Fig — “C1” and “C2” refer to samplings performed before and after acidification, respectively. The x-axis (in log-scale) represents the number of hours after the artificial dust deposition at the surface of both minicosms represented as a vertical dashed line. The error bars correspond to the sum of the sampling and analytical errors for each point. (TIF) [file pone.0171980.s002.tif]
